# 2-Amino-5-bromo­pyridinium 6-oxo-1,6-dihydro­pyridine-2-carboxyl­ate monohydrate

**DOI:** 10.1107/S1600536810030916

**Published:** 2010-08-11

**Authors:** Madhukar Hemamalini, Hoong-Kun Fun

**Affiliations:** a X-ray Crystallography Unit, School of Physics, Universiti Sains Malaysia, 11800 USM, Penang, Malaysia

## Abstract

In the crystal structure of the title salt, C_5_H_6_BrN_2_^+^·C_6_H_4_NO_3_^−^·H_2_O, the protonated N atom and the 2-amino group of the cation are hydrogen bonded to the carboxyl­ate O atoms of the anion *via* a pair of N—H⋯O hydrogen bonds, forming an *R*_2_^2^(8) ring motif. The ion pairs are further connected *via* O—H⋯O, N—H⋯O, N—H⋯Br and C—H⋯O hydrogen bonds, forming a two-dimensional network parallel to the *bc* plane. The water mol­ecules self-assemble through O—H⋯O hydrogen bonds, forming one-dimensional supra­molecular chains along the *a* axis, with graph-set notation *C*_2_^2^(4).

## Related literature

For background to the chemistry of substituted pyridines, see: Pozharski *et al.* (1997 ▶); Katritzky *et al.* (1996 ▶). For details of 6-hy­droxy­picolinic acid, see: Sun *et al.* (2004 ▶); Soares-Santos *et al.* (2003 ▶). For a related structure, see: Sawada & Ohashi (1998 ▶). For details of hydrogen bonding, see: Jeffrey & Saenger (1991 ▶); Jeffrey (1997 ▶); Scheiner (1997 ▶). For hydrogen-bond motifs, see: Bernstein *et al.* (1995 ▶).
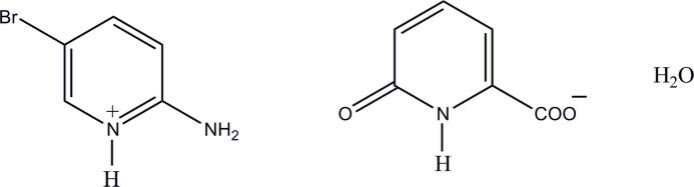


## Experimental

### 

#### Crystal data

C_5_H_6_BrN_2_^+^·C_6_H_4_NO_3_^−^·H_2_O
*M* *_r_* = 330.15
Orthorhombic, 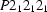

*a* = 3.8616 (1) Å
*b* = 15.8227 (2) Å
*c* = 20.8961 (3) Å
*V* = 1276.77 (4) Å^3^
*Z* = 4
Mo *K*α radiation
μ = 3.23 mm^−1^
*T* = 296 K
0.35 × 0.18 × 0.12 mm

#### Data collection

Bruker SMART APEXII CCD area-detector diffractometer
Absorption correction: multi-scan (*SADABS*; Bruker, 2009 ▶) *T*_min_ = 0.400, *T*_max_ = 0.694
8884 measured reflections
3718 independent reflections
3105 reflections with *I* > 2σ(*I*)
*R*_int_ = 0.022

#### Refinement

*R*[*F* ^2^ > 2σ(*F* ^2^)] = 0.031
*wR*(*F* ^2^) = 0.097
*S* = 1.09
3718 reflections
172 parameters
3 restraints
H-atom parameters constrained
Δρ_max_ = 0.37 e Å^−3^
Δρ_min_ = −0.32 e Å^−3^
Absolute structure: Flack (1983 ▶), 1482 Friedel pairs
Flack parameter: 0.011 (12)

### 

Data collection: *APEX2* (Bruker, 2009 ▶); cell refinement: *SAINT* (Bruker, 2009 ▶); data reduction: *SAINT*; program(s) used to solve structure: *SHELXTL* (Sheldrick, 2008 ▶); program(s) used to refine structure: *SHELXTL*; molecular graphics: *SHELXTL*; software used to prepare material for publication: *SHELXTL* and *PLATON* (Spek, 2009 ▶).

## Supplementary Material

Crystal structure: contains datablocks global, I. DOI: 10.1107/S1600536810030916/is2585sup1.cif

Structure factors: contains datablocks I. DOI: 10.1107/S1600536810030916/is2585Isup2.hkl

Additional supplementary materials:  crystallographic information; 3D view; checkCIF report

## Figures and Tables

**Table 1:** Hydrogen-bond geometry (Å, °)

*D*—H⋯*A*	*D*—H	H⋯*A*	*D*⋯*A*	*D*—H⋯*A*
N1—H1*B*⋯O2^i^	0.86	1.79	2.640 (4)	171
O1*W*—H1*W*⋯O2^ii^	0.94	2.17	2.730 (5)	117
N2—H2*A*⋯O3^i^	0.86	2.04	2.896 (4)	172
N2—H2*B*⋯O1^iii^	0.86	1.96	2.819 (4)	173
O1*W*—H2*W*⋯O1*W*^ii^	0.94	2.02	2.782 (9)	137
N3—H3*B*⋯Br1	0.86	2.84	3.681 (3)	168
C3—H3*A*⋯O1	0.93	2.44	3.351 (4)	167

Symmetry codes: (i) 
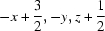
; (ii) 
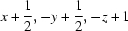
; (iii) 
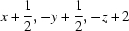
.

**Fig. 1.**
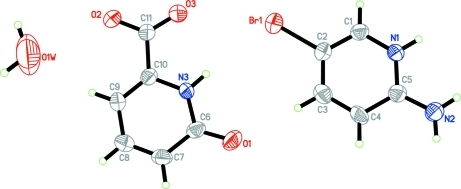
The asymmetric unit of the title compound. Displacement ellipsoids are drawn at the 50% probability level.

**Fig. 2.**
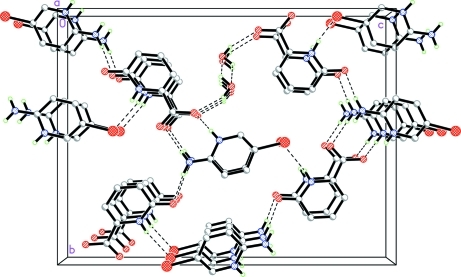
The crystal packing of (I), showing hydrogen-bonded (dashed lines) 2D networks parallel to the bc-plane. H atoms not involved in the intermolecular interactions have been omitted for clarity.

**Fig. 3.**
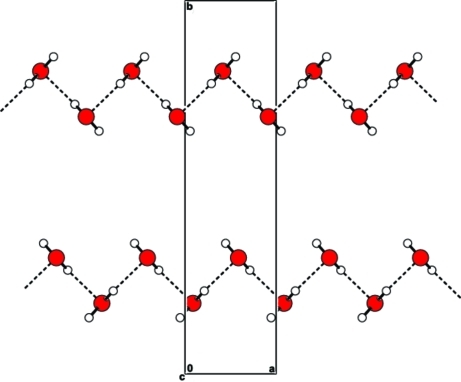
One-dimensional supramolecular chain made up of water molecules.

## supplementary crystallographic information

### Comment

Pyridine and its derivatives play an important role in heterocyclic chemistry (Pozharski *et al.*, 1997; Katritzky *et al.*, 1996). They are often involved in hydrogen-bond interactions (Jeffrey & Saenger, 1991; Jeffrey, 1997; Scheiner, 1997). 6-hydroxypioclinic acid has interesting characteristics:  firstly, it was characterized by a similar enol-keto tautomerism due to the labile hydrogen atom of -OH group in α-position migrating easily to the basic pyridine N atom; secondly, the multiple coordination sites such as the carbonyl oxygen, the amide nitrogen and carboxylate oxygen atoms are able to coordinate with various metal ions (Sun *et al.*, 2004; Soares-Santos *et al.*, 2003). In order to study some interesting hydrogen bonding interactions of these compounds, the synthesis and structure of the title salt is presented here.

The asymmetric unit, (Fig. 1), contains a 2-amino-5-bromopyridinium cation, a 6-oxo-1,6-dihydropyridine-2-carboxylate anion and a water molecule. The 2-amino-5-bromopyridinium cation is essentially planar, with a maximum deviation of 0.019 (3) Å for atom N1. In the 2-amino-5-bromopyridinium cation, a wider than normal angle [C1—N1—C5 = 122.7 (3)°] is subtented at the protonated N1 atom.  The anion exists in the keto-enol tautomerism of the -CONH moiety.  Similar form is also observed in the crystal structure of 2-oxo-1,2-dihydropyridine-6-carboxylic acid (Sawada & Ohashi, 1998).

In the crystal packing, (Fig. 2), the protonated N1 atom and the 2-amino group (N2) are hydrogen-bonded to the carboxylate oxygen atoms (O2 and O3) *via* a pair of intermolecular N—H···O hydrogen bonds, forming a ring motif *R*_2_^2^(8) (Bernstein *et al.*, 1995). The ion pairs are further connected via O—H···O, N—H···O, N—H···Br and C—H···O (Table 1) hydrogen bonds, forming a two-dimensional network parallel to the *bc* plane.  The water molecules self-assemble through O1W—H2W···O1W hydrogen bonds, forming one-dimensional supramolecular chains along the *a* axis, with graph-set notation *C*_2_^2^(4) (Fig. 3).

### Experimental

A hot methanol solution (20 ml) of 2-amino-5-bromopyridine (86 mg, Aldrich) and 6-hydroxypicolinic acid (69 mg, Merck) were mixed and warmed over a heating magnetic stirrer hotplate for a few minutes. The resulting solution was allowed to cool slowly at room temperature and crystals of the title compound appeared after a few days.

### Refinement

All hydrogen atoms were positioned geometrically (C—H = 0.93 Å, N—H = 0.86 Å and O—H = 0.9404–0.9428 Å) and were refined using a riding model, with *U*_iso_(H) = 1.2*U*_eq_(C, N, O).  1482 Friedel pairs were used to determine the absolute configuration.

### Crystal data

**Table:**

C_5_H_6_BrN_2_^+^·C_6_H_4_NO_3_^−^·H_2_O	*F*(000) = 664
*M**_r_* = 330.15	*D*_x_ = 1.718 Mg m^−^^3^
Orthorhombic, *P*2_1_2_1_2_1_	Mo *K*α radiation, λ = 0.71073 Å
Hall symbol: P 2ac 2ab	Cell parameters from 4297 reflections
*a* = 3.8616 (1) Å	θ = 2.6–27.5°
*b* = 15.8227 (2) Å	µ = 3.23 mm^−^^1^
*c* = 20.8961 (3) Å	*T* = 296 K
*V* = 1276.77 (4) Å^3^	Block, colourless
*Z* = 4	0.35 × 0.18 × 0.12 mm

### Data collection

**Table:**

Bruker SMART APEXII CCD area-detector diffractometer	3718 independent reflections
Radiation source: fine-focus sealed tube	3105 reflections with *I* > 2σ(*I*)
graphite	*R*_int_ = 0.022
φ and ω scans	θ_max_ = 30.1°, θ_min_ = 1.6°
Absorption correction: multi-scan (*SADABS*; Bruker, 2009)	*h* = −5→5
*T*_min_ = 0.400, *T*_max_ = 0.694	*k* = −22→19
8884 measured reflections	*l* = −20→29

### Refinement

**Table:**

Refinement on *F*^2^	Secondary atom site location: difference Fourier map
Least-squares matrix: full	Hydrogen site location: inferred from neighbouring sites
*R*[*F*^2^ > 2σ(*F*^2^)] = 0.031	H-atom parameters constrained
*wR*(*F*^2^) = 0.097	*w* = 1/[σ^2^(*F*_o_^2^) + (0.036*P*)^2^ + 0.5546*P*] where *P* = (*F*_o_^2^ + 2*F*_c_^2^)/3
*S* = 1.09	(Δ/σ)_max_ = 0.001
3718 reflections	Δρ_max_ = 0.37 e Å^−^^3^
172 parameters	Δρ_min_ = −0.32 e Å^−^^3^
3 restraints	Absolute structure: Flack (1983), 1482 Friedel pairs
Primary atom site location: structure-invariant direct methods	Flack parameter: 0.011 (12)

### Special details

**Table:**

Geometry. All s.u.'s (except the s.u. in the dihedral angle between two l.s. planes) are estimated using the full covariance matrix. The cell s.u.'s are taken into account individually in the estimation of s.u.'s in distances, angles and torsion angles; correlations between s.u.'s in cell parameters are only used when they are defined by crystal symmetry. An approximate (isotropic) treatment of cell s.u.'s is used for estimating s.u.'s involving l.s. planes.
Refinement. Refinement of F^2^ against ALL reflections. The weighted R-factor wR and goodness of fit S are based on F^2^, conventional R-factors R are based on F, with F set to zero for negative F^2^. The threshold expression of F^2^ > 2σ(F^2^) is used only for calculating R-factors(gt) etc. and is not relevant to the choice of reflections for refinement. R-factors based on F^2^ are statistically about twice as large as those based on F, and R- factors based on ALL data will be even larger.

### Fractional atomic coordinates and isotropic or equivalent isotropic displacement parameters (Å^2^)

**Table:**

	*x*	*y*	*z*	*U*_iso_*/*U*_eq_	
Br1	0.68523 (9)	0.02155 (2)	0.832419 (15)	0.04315 (11)	
N1	0.9529 (7)	0.01356 (17)	1.02199 (12)	0.0358 (5)	
H1B	1.0573	−0.0221	1.0463	0.043*	
N2	0.8795 (10)	0.09857 (18)	1.10959 (13)	0.0490 (8)	
H2A	0.9800	0.0609	1.1327	0.059*	
H2B	0.8067	0.1446	1.1268	0.059*	
C1	0.9123 (9)	−0.00566 (19)	0.95935 (15)	0.0357 (7)	
H1A	0.9939	−0.0570	0.9437	0.043*	
C2	0.7526 (7)	0.04972 (19)	0.91899 (14)	0.0347 (7)	
C3	0.6342 (9)	0.1270 (2)	0.94344 (16)	0.0408 (7)	
H3A	0.5271	0.1657	0.9164	0.049*	
C4	0.6750 (10)	0.14591 (18)	1.00655 (15)	0.0384 (7)	
H4A	0.5981	0.1974	1.0227	0.046*	
C5	0.8371 (10)	0.08579 (19)	1.04763 (14)	0.0363 (6)	
O1	0.1449 (9)	0.24380 (15)	0.84486 (11)	0.0517 (7)	
O2	0.1646 (8)	0.08864 (15)	0.59255 (11)	0.0525 (6)	
O3	0.3456 (8)	0.03807 (15)	0.68650 (11)	0.0529 (7)	
N3	0.1458 (8)	0.17784 (15)	0.74829 (11)	0.0337 (5)	
H3B	0.2465	0.1354	0.7660	0.040*	
C6	0.0674 (9)	0.24462 (19)	0.78634 (16)	0.0374 (7)	
C7	−0.0997 (9)	0.3135 (2)	0.75378 (17)	0.0427 (8)	
H7A	−0.1583	0.3621	0.7764	0.051*	
C8	−0.1722 (11)	0.3084 (2)	0.69074 (17)	0.0438 (7)	
H8A	−0.2841	0.3532	0.6708	0.053*	
C9	−0.0818 (10)	0.2363 (2)	0.65430 (15)	0.0398 (7)	
H9A	−0.1321	0.2333	0.6108	0.048*	
C10	0.0787 (9)	0.1721 (2)	0.68434 (14)	0.0351 (7)	
C11	0.2065 (10)	0.09186 (19)	0.65253 (14)	0.0378 (7)	
O1W	0.5862 (19)	0.3110 (3)	0.51273 (19)	0.133 (2)	
H1W	0.4451	0.3496	0.4906	0.159*	
H2W	0.7111	0.2778	0.4832	0.159*	

### Atomic displacement parameters (Å^2^)

**Table:**

	*U*^11^	*U*^22^	*U*^33^	*U*^12^	*U*^13^	*U*^23^
Br1	0.04628 (18)	0.04800 (18)	0.03519 (15)	−0.00207 (15)	−0.00408 (15)	−0.00074 (15)
N1	0.0457 (14)	0.0297 (12)	0.0319 (11)	0.0062 (12)	−0.0001 (11)	0.0035 (11)
N2	0.076 (2)	0.0353 (13)	0.0355 (13)	0.0094 (15)	−0.0032 (15)	0.0006 (12)
C1	0.0412 (16)	0.0307 (15)	0.0353 (14)	0.0010 (12)	0.0025 (13)	−0.0024 (12)
C2	0.0359 (19)	0.0369 (14)	0.0312 (13)	−0.0034 (12)	0.0007 (12)	−0.0003 (12)
C3	0.0340 (18)	0.0442 (17)	0.0443 (16)	0.0034 (14)	−0.0016 (15)	0.0061 (14)
C4	0.0444 (16)	0.0276 (13)	0.0432 (16)	0.0049 (14)	0.0015 (17)	0.0124 (12)
C5	0.0387 (16)	0.0338 (14)	0.0365 (14)	0.0010 (14)	0.0007 (15)	−0.0001 (12)
O1	0.0781 (19)	0.0436 (13)	0.0334 (11)	−0.0015 (14)	−0.0032 (13)	−0.0099 (10)
O2	0.0727 (17)	0.0530 (13)	0.0318 (10)	0.0202 (14)	−0.0018 (13)	−0.0070 (10)
O3	0.0790 (19)	0.0410 (13)	0.0388 (11)	0.0190 (13)	−0.0049 (13)	−0.0051 (10)
N3	0.0465 (15)	0.0257 (11)	0.0288 (11)	0.0039 (11)	−0.0007 (12)	0.0035 (9)
C6	0.0464 (18)	0.0300 (15)	0.0357 (15)	−0.0040 (13)	0.0034 (14)	−0.0069 (13)
C7	0.044 (2)	0.0346 (16)	0.0492 (18)	0.0092 (14)	0.0090 (16)	−0.0031 (14)
C8	0.0439 (18)	0.0401 (16)	0.0475 (17)	0.0097 (17)	0.0011 (17)	0.0005 (14)
C9	0.0464 (18)	0.0403 (17)	0.0327 (15)	0.0047 (14)	0.0000 (13)	0.0055 (13)
C10	0.0374 (16)	0.0370 (15)	0.0308 (14)	0.0004 (13)	0.0030 (12)	−0.0009 (12)
C11	0.0475 (18)	0.0330 (14)	0.0329 (14)	0.0045 (14)	0.0000 (14)	−0.0007 (11)
O1W	0.216 (7)	0.109 (3)	0.074 (2)	−0.002 (4)	−0.033 (4)	0.026 (2)

### Geometric parameters (Å, °)

**Table:**

Br1—C2	1.881 (3)	O2—C11	1.265 (4)
N1—C5	1.339 (4)	O3—C11	1.231 (4)
N1—C1	1.353 (4)	N3—C6	1.356 (4)
N1—H1B	0.8600	N3—C10	1.364 (4)
N2—C5	1.321 (4)	N3—H3B	0.8600
N2—H2A	0.8600	C6—C7	1.438 (5)
N2—H2B	0.8600	C7—C8	1.349 (5)
C1—C2	1.364 (4)	C7—H7A	0.9300
C1—H1A	0.9300	C8—C9	1.415 (5)
C2—C3	1.402 (5)	C8—H8A	0.9300
C3—C4	1.361 (5)	C9—C10	1.345 (5)
C3—H3A	0.9300	C9—H9A	0.9300
C4—C5	1.426 (4)	C10—C11	1.516 (4)
C4—H4A	0.9300	O1W—H1W	0.9404
O1—C6	1.259 (4)	O1W—H2W	0.9428
			
C5—N1—C1	122.7 (3)	C6—N3—H3B	117.2
C5—N1—H1B	118.6	C10—N3—H3B	117.2
C1—N1—H1B	118.6	O1—C6—N3	120.5 (3)
C5—N2—H2A	120.0	O1—C6—C7	125.0 (3)
C5—N2—H2B	120.0	N3—C6—C7	114.4 (3)
H2A—N2—H2B	120.0	C8—C7—C6	120.6 (3)
N1—C1—C2	120.4 (3)	C8—C7—H7A	119.7
N1—C1—H1A	119.8	C6—C7—H7A	119.7
C2—C1—H1A	119.8	C7—C8—C9	121.5 (3)
C1—C2—C3	118.9 (3)	C7—C8—H8A	119.2
C1—C2—Br1	120.3 (2)	C9—C8—H8A	119.2
C3—C2—Br1	120.8 (2)	C10—C9—C8	118.1 (3)
C4—C3—C2	120.4 (3)	C10—C9—H9A	121.0
C4—C3—H3A	119.8	C8—C9—H9A	121.0
C2—C3—H3A	119.8	C9—C10—N3	119.7 (3)
C3—C4—C5	119.2 (3)	C9—C10—C11	125.3 (3)
C3—C4—H4A	120.4	N3—C10—C11	115.0 (3)
C5—C4—H4A	120.4	O3—C11—O2	126.8 (3)
N2—C5—N1	118.8 (3)	O3—C11—C10	117.9 (3)
N2—C5—C4	122.9 (3)	O2—C11—C10	115.3 (3)
N1—C5—C4	118.4 (3)	H1W—O1W—H2W	109.7
C6—N3—C10	125.7 (3)		
			
C5—N1—C1—C2	0.9 (5)	O1—C6—C7—C8	180.0 (4)
N1—C1—C2—C3	0.6 (5)	N3—C6—C7—C8	1.1 (5)
N1—C1—C2—Br1	−178.3 (2)	C6—C7—C8—C9	−1.1 (6)
C1—C2—C3—C4	−0.7 (5)	C7—C8—C9—C10	0.2 (6)
Br1—C2—C3—C4	178.1 (3)	C8—C9—C10—N3	0.7 (5)
C2—C3—C4—C5	−0.5 (5)	C8—C9—C10—C11	−177.5 (3)
C1—N1—C5—N2	178.2 (3)	C6—N3—C10—C9	−0.8 (5)
C1—N1—C5—C4	−2.2 (5)	C6—N3—C10—C11	177.6 (3)
C3—C4—C5—N2	−178.5 (4)	C9—C10—C11—O3	−179.1 (4)
C3—C4—C5—N1	1.9 (5)	N3—C10—C11—O3	2.6 (5)
C10—N3—C6—O1	−179.1 (4)	C9—C10—C11—O2	2.6 (6)
C10—N3—C6—C7	−0.1 (5)	N3—C10—C11—O2	−175.7 (3)

### Hydrogen-bond geometry (Å, °)

**Table:**

*D*—H···*A*	*D*—H	H···*A*	*D*···*A*	*D*—H···*A*
N1—H1B···O2^i^	0.86	1.79	2.640 (4)	171.
O1W—H1W···O2^ii^	0.94	2.17	2.730 (5)	117.
N2—H2A···O3^i^	0.86	2.04	2.896 (4)	172.
N2—H2B···O1^iii^	0.86	1.96	2.819 (4)	173.
O1W—H2W···O1W^ii^	0.94	2.02	2.782 (9)	137.
N3—H3B···Br1	0.86	2.84	3.681 (3)	168.
C3—H3A···O1	0.93	2.44	3.351 (4)	167.

Symmetry codes: (i) −*x*+3/2, −*y*, *z*+1/2; (ii) *x*+1/2, −*y*+1/2, −*z*+1; (iii) *x*+1/2, −*y*+1/2, −*z*+2.
